# Supplementary material for: Association Mapping of Verticillium Wilt Disease in a Worldwide Collection of Cotton (Gossypium hirsutum L.)
Source: Plants (Basel). 2021 Feb 5;10(2):306. doi: 10.3390/plants10020306 (PMC7916069; doi:10.3390/plants10020306)
Supplement: Supplementary file 1 [file plants-10-00306-s001.pdf]

Supplementary Materials Table S1. Sequences of markers showing significant association

Marker	Consensus_Seq	Position on Consensus	Ch.	Posiotion on Chromosome	Gene
A3190	CAGCTATGTCACCAAAAGCCCAAATCAAGCCTGAGCTTAAAACCTGTGTCTTMACTGGGTGGATTGCTAAGCAATTTTGATA	53	3	61.076.712	LOC107948714 protein Mpv17-like
A939	CAGCATATTCAAAGAATCTGGTAAATTATTTTTGTTGTCAATACAAGTTTAATACCTGGKAGTGGTGGGAGAAAAGAAAGAT	60	D11	42.444.312	NA
A5075	CAGCTTTTGTCTCTAACCTCTCCCGTGAAGCAGACTACGGTGCCGACCCACGAGCCAGCYCCGCCTTACACGACTGTTTCTC	60	19-25	12.564.707- 19.945.402	LOC107903729, LOC107921167, 21 kDa protein-like
A4529	CAGCTAAGTCCTTCAAACCCAAATAACATAACAATGCATTCATATATGGAGKTGAAATATTCTGATTGTTACCACTTAAGGA	52	D11	36.723.905	LOC105804552 transcription factor MYB113-like
A7660	CAGCAGAGGCAGACAAAYTTTGCAAGGCTTTTCAACACGTTCACAGGAAACTTGTATGTCTATTTTAGTTAATCCCACATAA	18	D9	29.949.760	NA
A2118	CAGCCGTGTTAGATTCTTTCGCTTTTACAATCAAACCCAGCTACAGTTTAGAGKGTTATCTAAGTTTCAATGGAGGACGATA	54	21-26	9.730.256- 20.661. 882	LOC107912355, LOC107924363 protein DEHYDRATION-INDUCED 19 homolog 3-like
A5526	CAGCCCAATCTTTGTGGTATTTTCTTATTTGCCTGCGCTAGAACCCGTAGGTGCACAAGTRCAAAATTTCTTGAGAAGTAAG	61	Na	3.027	LOC107941261
A8104	CAGCAGTGGAGAAAGCTTTTGGTCTCATGAATGGGAGAAACACGGTACCTGCTCCGAGTCCCTCCTTGATCAACAYAGCTAC	76	2-24	102.210.077- 45.178.035	LOC107902274, LOC107919674 extracellular ribonuclease LE-like
A464	CAGCCAATAACTAGAGAAKCTGAACCCTGCATATGAACATAATCATCAAAAACCGACCTATGAACCTTGTATAATTCTATTC	19	D4	44.272.173	LOC107944641 F-box protein CPR30-like
A9833	CAGCATTATACTTCCTAGACCAACTTTTAACTGGTTTTAATGAAGATAGAAACTTAGAGAAYGCTTGCCAAGTGGTTTCTCT	62	22-16	60.934.191- 63.975.517	LOC107897477 putative E3 ubiquitin-protein ligase LIN
A8451	CAGCAGATGAGTATGTGACGATGGCCAAACGATCAATTGGTCGGAGGGAGAATACCACTAAYGCCATGGACTGTTTTAGAAG	62	12-10	1.220.085- 83.702.765	LOC107963741-LOC107960135
A9533	CAGCATGTTGTTCATAAGGTCAGTCTGGATGCTCTTATTTCACAGTTRGAATTAAATATAAATGGTCTTTAAGGTACTGTGA	48	D8	48.778.681	
A7122	CAGCTGCATTTATAATAGGGCCARTCTTTAGAACCGGCAGATCACGCCGTCAAACCAGATGAGTATAGTACCCTTAGCAATT	24	D3	42.351.83	
A9046	CAGCAACTTCACCAATCTTAACATTTCCATGGAGTTTACAAGMACCCAATAAAGCTCTCCATATTACTGAATTGGCCTCAAA	43	17	25.971.276	LOC107899479 (pentatricopeptide repeat-containing protein At3g62890-like)
A3886	CAGCACCGGTGGTGTATTGCCAAAATGTGTTGAATAATACGAAAACAGTATGTYGGATTGGCTATCTTGCGGATTTTTTCTT	54	D9	7.626.784	
A2768	CAGCATCCGAATCATCTTCCTCAAACTTTGATGTAAATAACCAAAGAAGCATSGGAGAGAGCAGAGATGTGTGGCCCAAAGC	53	24	30.431.399	LOC107918458
A9566	CAGCCAACTTATTGAATTATCACCTACATATTTGGATATTTTGCAATGCKACGAAGAATTTAATAACTAAAAGTCTAAAAGG	50	D9	11.264.238	
A4120	CAGCATTCGATGAATTTCACCAACAAGGCAACCTATGTAGCGGCCTGAGTATGGTTTGCCATTGGATCCTGGTTGCCTMAGG	79	6-17	67.041.464- 9.190.733	LOC107948854-LOC107899022 (fructose-1,6-bisphosphatase, chloroplastic-like)
A5502	CAGCTTTCATCATGTTGATTAATGGTTTAGAAAAATCATGAATAGCTTTGTAAGCAGATTCAACARCTGATGTGAAGAGCTC	66	21-D11	2450321- 3328322	LOC107911588 IRK-interacting protein-like, transcript variant X2
A5821	CAGCAGCRATAGAAGCATATGAAAAAGATTTTAAAAACCTTTCATATTATGCTGACTTCTTCCTTAATTGGAAACATGAAAA	8	D5	29.389.195	
A4657	CAGCTGGTGGAAAAGCAATATGCCCTAAAACCAGATTGCAGATCAGCCGRCAAAAACTTCTCAAAGTACCAGCTCTAACATC	50	12	9.125.748	LOC105780137 glycosylphosphatidylinositol anchor attachment 1 protein
A5901	CAGCATGTACGTACCCAAATAAGTTTGTGGCCGACACAACTACGACAGAAGCGAGAGCCTGTTTGCAAKCTGAGGTGGGAAA	69	Na	Na	

A4075	CAGCATGAGATGGAGAACCAACYCCACTGTGGAGATCTTCGAAAGGCTTCACATTCACAAACACAATTAGACATAGGCGTAT	23	D4	13.047.156	
A8965	CAGCCCAAGTGAGAGGTTAGATTCAACTTGAATGAGCAYTTTAAAAGAAATTCACTAAGTAGAACTCAGAGTATTACTTATA	39	D6	18.954.790	
A558	CAGCTTCGACATGCCTCGGCTATACCTACGCTCATTCTCCACATCACCAGTYGTAGAAAATGTCTCACTTGGTTAGGCACAT	52	D10	53.314.674	
A7246	CAGCAATACATGTTAGTGAATGTGTATATATATGTACCTTGGGGCTTTGRCCCTTGGCAAGCTCAGCATACAAAGCAGGATT	50	D4	42.119.775	
A339	CAGCATGTTTGAGCGTTTACAAAGAGCTGGTCATCCTGTTGTTATGCTTACTGAACAGGTARCCTTCACTGCAATTGCTTAT	62	D6	27.507.643	
A2168	CAGCATCCAATTACCCCATTTGGAGTCCCACTTTAACGTTAACTATATGTTGTTACAATGGGGGTGGKAGGGTTGGGGGATC	68	16	24.453.514	LOC107896835 U3 small nucleolar RNA-associated protein 4-like
A7785	CAGCTGATCATCGGAGAGTCCGTGTTTCTGTTASCCTGAATTTGTTTTTTATAGGAGCTTGTGGATTGGGAACATAGGAGCA	34	D4	158.221	
A7267	CAGCAGATTGTTGTTCTTTGACAGCCTCCTCCATGATTGTTCGTTTCTCTTCCAAGATTTTTCTCAAACAAAAAGAAAAAKA	81	5	4.645.398	LOC107946501 microtubule-associated protein futsch-like
A5067	CAGCTTAGCAATCAGCAAGGTCCTTCCTTGCAAAATATAACATTTTATCAGGCACTCGGYCATACATCACTCCATTTATAAC	60	25	42.960.772	LOC107921815 transport and Golgi organization 2 homolog
A2316	CAGCAGTCGACAGGATTACTCTGAAATGACTCAAATCAAGTATCTCCTTAATGAAATGGAATTGTCTGGTAAAAAAAAAAAR	82	D1	51.853.370	
A5343	CAGCATTAAATACCTTTTAAAGCTCATTTTGCCTGCATGTTTGTGCCAGTTTTTSAAGTTTTGCAGTGTGGATTCCTTAGTT	55	D9	39.774.356	
A1772	CAGCTTCTTTTCACTTTGATGTGCCTGAAAYATACAGCAGGATCTCGAAACTAAAGAAGATGAAAGCTTCTCTAAGACATTA	31	1	87.736.674	LOC107951191 protein NETWORKED 1D-like
A6025	CTGCCTCATTTTCATCTGATCAGTGTGCTGAAGGTGTTGTTGCTGTTGCTGGGGATGCATTGAGGGTTTTCACCATYGAGCG	77	10	66.517.458	LOC107959892 splicing factor 3B subunit 3-like
A680	CAGCTTGAAAAGTTGAAAATATTCAAGACCATGTTGGAGCGCATCATAGGTTTCTTAACAGTTTCCAGAGYCAATATAACAC	71	22-16	6355122-57.747.916	LOC107914147 mediator of RNA polymerase II transcription subunit 15a-like, LOC107897284
A6147	CAGCGATCAACGGGAGAGATCAACATGGATGGACCGCATTACATCGAGCATCGTTTAAAGGACGAACGGATGCCRTTAAGAT	75	4-15	76412909-21.225.148	LOC107937316 putative ankyrin repeat protein RF_0381, LOC107893150 ankyrin-3-like
A4041	CAGCACACAGCCCAGTATTKATGAAAGTATCCAAAACTCGAAGTCAGGTAATTTCATTCCTTCTTCTACTTCCTTTTTAAAT	20	D6	13.120.530	
A306	CTGCTCCTCAAGCCTTCGAGTGCCGATCTTCGCTTGGGCCGGCTCCGAGGCTCTACCCTGGCTTTTCATTGGTTCCAACSCT	80	Mt JX944505.1	202658-656946	
A5072	CAGCTTCAGCTCCACTTCTAAAACGCCATCAACTTCATGGGAATTGCAACTAACAAAKTCATTGATTGTCATAATGGATTTC	58	D11	4.784.898	
A412	CAGCCACTGGCACATAATCAGCGACAGTATTATAATCAGAAATCTTCCCAAMCGGCATTGCAATTGAACTGACTCCAGAAAT	52	D1&XM_016856208.1	7438178-368	LOC107925491 tubby-like F-box protein 8 , transcript variant X2
A6306	CAGCTTATTACACGAATACATCGAAAGTTCACTGCAAACTACTTTTGGKTCGAGAGATACTGCTAAAACTCAATGTGCTGTG	49	D2	36.632.816	
A4574	CAGCTTTGAGTTCCTTTTCGTAAGCAATGGCGGACCAAGCCAAGGTAGTGATCGTAAACGCMATCGGCATCCCGTATTTCAC	62	D8	61.214.324	LOC107932833 endoglucanase 16-like
A5855	CAGCTAAAAGGATTAAGATTGGGATTTACTTTGCTACTTGGTGGGCTKTGAATGTAGTTTTCAACATATATAACAAGAAGGT	48	18	49.931.223	LOC107901936 glucose-6-phosphate/phosphate translocator 2, chloroplastic-like

A4939	CAGCAGTCATCTATTGTCAGCAAAGGTGGAACTATGATCAGCTTAGCCAGATTCTGACMTTCAAGCAATGTAGGCTTCTTCC	59	16	56.101.995	LOC107897225
A9364	CAGCAAAAAGAACCATGTTTGCAACATTGGAAATTCTAATGGCTAACGTCTCACTTCTAGCCAACYTTTCCCTTTCTTCCTG	66	16	90.067.393	LOC107897780 metal tolerance protein 11-like
A1641	CAGCACCTTGATACACAAAATATGATCCTCTATGTTTATAATTTGTTCAAGTMTGTGCTTTAGATGGTAATAAATTACTCTC	53	D13	51.408.054	
A2970	CAGCTGTAGTTCACCTGCAAGAAGTCCATGTAATCCATTCGGTATAGATGATMGCCAGATAATATCAGAATGTTCTCAATAT	53	D7	33.833.694	
A1365	CAGCCCAAACCATCCCATCTTGCTGAAAAAATCAGCTAAATTTATCTTATTTTAACACTTACAAAATAKCCCTTAAACTCTC	69	Na		
A9109	CAGCAGGAGAAAGTTGCACACGAGGTTTTCTCCTGAGACGCTTAGGGAAGATGGTATTAAGAAGTTTAGGCTTYGAGTGCTG	74	D2	46.908.558	
A3946	CAGCGGCATCAATAATTCTCACTTTTCATGACCACATTGATTTAAAAAMGAAAAAAGAAAAAGAAAAAGAGAACAATAACAT	49	Na		
A1595	CAGCTTCACGCATTGCTTGCAACTCAGCCTATAGAAAGAAAAGGATTAATATAMTAAGTTGATACTAATAAGTTCATCAAAT	54	NA		
A4814	CAGCAAAATCAGGGTAAAGTTGAGTTCAATAGTTAAGCAAGATTGTGTTTTMATTAAGGGAGAGAGAGAAACCCATTTTTCT	52	D9	20.084.101	
A968	CAGCGTATGGATTATATTGACAGGAATGATAAAAATCAATTACTTCTTGGCATCCGGCGAGCWAATCGACCTCAAACTGTAA	63	3	27.667.043	LOC107914851 VAN3-binding protein-like
A7740	CAGCCTCATGTGCCTGTAATCATGGAAGCTTGCATGAAAAATGCTACTGAAGTTGAGAAGCCTCTTSGTTATCTCCAACTTC	67	D11-21	13160961-21.368.426	LOC107912994 transformation/transcription domain-associated protein-like
A1176	CAGCGTCATCTCAAGTACTGGATTCCGGAAACGGACAGACCGGAGTTTYGGGGGTTTTATCAGCGGACAACGTGGTGGCGGT	49	Na	Na	LOC107929115 pyruvate kinase isozyme A, chloroplastic-like
A4948	CAGCAAGAATCCTCCCGAGGTTCGCTCTCGATTTCTCGAGATTCTTTGACGCYTTGCGACGAATCGACTTCGTCGGTCGAAA	53	1	27.108.899	LOC107920268 ethylene-responsive transcription factor CRF2-like
A5085	CAGCAATCCCAATACTTATTTAACTATCAACTCTTTTTTTTTATTCATMATCATCATCAAAGGTCATCAAATCTAAAAATAA	49	Na	Na	
A3067	CAGCCGTCCTCTTCCTTCTCGGGGCTACCGGTCTGATGTCGACCMTTTACCGTCTCCTCCCTCTCCCTGTTGTCCGCGGGAT	45	16	56.089.930	LOC107897224 molybdate transporter 2-like
A2219	CAGCTTGGACTTCACTCTGAAAAGGCAAGTTGACATKTCATATATACTGCTTAAACAAATTAGTATGCTATAATCATTGAGA	37	D6	25.619.204	
A7299	CAGCTTGAATAATGGCAGAAAGCTTAAAGTTTACATCTCCTCCGTGAAGGCCATAKATGTACTTCTAGAAAGAAAAATCATA	56	D7	13.842.749	
A1756	CAGCGTAGGTTATTGCCTAAAMTTGAAATGGTATCACCTAATGGACCATTCAAGTCCCTTGAAGAACTATGGGATGGGGAGA	22	Na	303.898	LOC107933372 non-functional NADPH-dependent codeinone reductase 2-like

Supplementary Table S2. Germplasm Collection

Genotype	Origin	Genotype	Origin	Genotype	Origin	Genotype	Origin
1118-Glandless	USA	Corina	Spain	Okra 204	USA	YB141	Turkey
152-F	Uzbekistan	Crincle Leaf	USA	Okra-frego	USA	Acala 44	USA
153-F	Uzbekistan	Cirpan 603	Bulgaria	P.D. 0648	USA	Acala Royale	USA
2421-A	Russia	Cukurova-1518	Turkey	Paymaster 2379	USA	Acala1517-99	USA
308 (CAMPO)	Greece	Cun S-1	USA.	Paymaster 330	USA	Acala Prema	USA
4SP	Albania	Delcerro	USA	R-5 (STG-6)	Turkey	Acala1517-95	USA
919 (LIDER)	Greece	Delta Opal	USA	RKNR 261	USA	Stoneville 132	USA
93 FF 01	USA	DP-388	USA	SAHEL 1	Iran	YB149	Turkey
Acala Maxa	USA	DPL-20	USA	SAYAR-314	Turkey	YB150	Turkey
Acala-172	USA	DPL-50	USA	Semer. Uzbek	Uzbekistan	YB151	Turkey
Acala-552	USA	DPL-5409	USA	Semu SS7G	Australia	YB152	Turkey
AK-4	Russia	DPL-5614	USA.	Sure Grow 404	USA	YB1535	Turkey
Aktas-3	Azerbaijan	AB80	Turkey	Sure Grow 501	USA	YB154	Turkey
Albania-6172	Albania	EUROPA-1752	Israel	Sindos 80	Greece	YB155	Turkey
Aleppo 1	Syria	Fibermax 819	USA	Siocra	Australia	YB156	Turkey
Aleppo 40	Syria	Fibermax 832	USA	Sivon	Isreal	YB157	Turkey
Aydın-110	Turkey	Fibermax 958	USA	Sphinx V	USA	YB158	Turkey
Azerbaycan 3038	Azerbhaican	Garant	Albania.	STG 14	USA	YB159	Turkey
Beli İzvor-432	Bulgaria	Gedera-5	Turkey	Stn 8a	USA	YB160	Turkey
Belserroms-30		Golda	Turkey	Stoneville-453	USA	YB161	Turkey
BSC-4	USA	Gurelbey (34/1)	Turkey	Suregrow 125	USA	Gosspollfree	Turkey
CA-228	Africa	IS-2	Israel	Sahin 2000	Turkey	PI 528420	USA
Carmen	Australia	Kahinath	India	Tamcot CABCS	USA	NP-ozbek 100	Turkey
Caskot BR-1	USA	Lachata	Spain	Tamcot Luxor	USA	TX 0175-2	USA
Maras92	Tukrey	H-88029	India	Tamcot Pyramid	USA	Özbek 105	Turkey
Marcel leaf	USA	Hint Ç.9	India	Tamcot SP 37-N	USA	TX 0175-1	USA
McNair-235-612	USA	HYC-76/59	Turkey	Tamcot Sphinx	USA	TX 0061-2	USA
MC NAMARA	USA	İs 4	Israel	Taskend-6	Uzbekistan	Nazilli 07	Turkey
NAKBC1-14/2	Turkey	İs 8	Israel	YB101	Turkey	Sezener 76	Turkey
NATA	Spain	Kurak-1	Turkey	TKY-9409	USA	TX 0060-2	USA
Nazilli 342	Turkey	Lockette		Togo	Africa	TX 0091-1	USA
Nazilli 84S	Turkey.	Nazilli 87	Turkey	Veramine	Iran	İpek 607	Turkey
Nazilli M-503	Turkey	Özbek 142	Turkey	Zeta 2	Greece	PI 528426	USA
Nazilli (93-7)	Turkey	Visalia Elmer		YB106	Turkey	NP EGE 2009	Turkey
Nectar free	Turkey.	Sealand 542	USA	Kurak 2	Turkey	PI 173332	USA
Nieves	Australia	Siokra 133	USA	NGF-63	Turkey	PI 529128	USA
NSCH-777	India	STN. K311	USA	Naked	Turkey	STN498	USA
Okra 201	USA	Stonville 506	USA	Orgosta 644	Bulgaria	TX 0091-2	USA

Samon	albanian	PI 165325	USA	İs 10	Israel	GAİA	Turkey
Ujchi 2 Uzbek	Uzbekistan	ZN243	Turkey	GSN 12	Turkey	ADN 710	Turkey
108F	Russia	PI 528429	USA	HT1	Turkey	TMN 16	Turkey
Acala 3080	USA	PI 528450	USA	Naz 143	Turkey	TMS 108/2	Turkey
Acala S.J. 2	USA	PI 528525	USA	Emand 542		ADN 712	Turkey
Coker 413/68	USA	GAPEAM1	Turkey	Flora	Australia	TMN 199	Turkey
DPL 15/21	USA	PI 529869	USA	Napa	Turkey	BEREN	Turkey
DPL529	USA	Spears3(967)	USA	YB247	Turkey	Sarı Gelin	Turkey
DPL 90	USA	YB193	Turkey	DP 493	Turkey	Nihal	Turkey
Ege-69	Turkey	YB194	Turkey	H- 23	Turkey	Gelincik	Turkey
Extreme Okra	Turkey	YB195	Turkey	GSN 22	Turkey	TMN 18	Turkey
Eksi-911	Turkey	YB196	Turkey	Celia	Australia	ADN 413	Turkey
Gossypollfree86	Turkey	YB198	Turkey	Cooker 100 A 2	USA	Ozaltın 112	Turkey
TX0175-1	USA	DP419	USA	Cabu cs 2-1-8-3	USA	Özaltın 404	Turkey
TX 0175-2	USA	Primera	USA	Menderes 2005	Turkey	Lodos	Turkey
528875	USA	Veret	Isreal	S-9	Syria	Flash	Turkey
Acala wild 1517	USA	BA 525	Turkey	H-10	Turkey	Carisma	Turkey
Ugur	Turkey	Delta Pine 5690	USA	DP 5111	USA	Aksel	Turkey
Acala 1517-99	USA	SJU 86	USA	SG 96	USA	BA 440	Turkey
TX 0091-2	USA	Blightmaster	USA	Adana 98	Turkey	BA 811	Turkey
YB214	Turkey	Sicala 33	Australia	Cun S-2	Turkey	Lydia	Turkey
YB215	Turkey	HT2	Turkey	Tamcot SP 21-9	USA	PG 2018	Turkey
YB216	Turkey	Dicle 2002	Turkey	Siokra L 22	Australia	Julia	Australia
PI 163722	USA	Semu 55/6	Australia	Coskun-1	Turkey	Claudia	Australia
PI 163615	USA	Tropical 225		DKG 658	Turkey	Carla	Australia
163615	USA	STV 373	USA	Naz M 39	Turkey	Candia	Australia
YB225	Turkey	Naz 84	Turkey	DP 396	USA	Gloria	Australia
Krem	Turkey	4 SB		Coker 312	USA	Nazilli 303	Turkey
Acala 1517 D	USA	İdeal		Sicala 3/2		Siokra 1/4	Australia
ADN 123	USA	Vurcano	Spain	Tamcot H 0 95	USA	YB289	USA
Sealand 1	USA	Stoneville 478	USA	Gossypolsüz Nazilli	Turkey	STV 474	USA
TMN 170	Turkey	Sure Grow 1001	USA	Cooker 100 Ahıl	USA	Fantom	Greece
TM-1	USA	Barut 2005	Turkey	Nazilli 954	Turkey	Famosa	USA
				Paymaster 404	USA	TMK 122	Turkey
